# Development of Human Adrenocortical Adenoma (HAA1) Cell Line from Zona Reticularis

**DOI:** 10.3390/ijms24010584

**Published:** 2022-12-29

**Authors:** Hans K. Ghayee, Yiling Xu, Heather Hatch, Richard Brockway, Asha S. Multani, Tongjun Gu, Wendy B. Bollag, Adina Turcu, William E. Rainey, Juilee Rege, Kazutaka Nanba, Vikash J. Bhagwandin, Fiemu Nwariaku, Victor Stastny, Adi F. Gazdar, Jerry W. Shay, Richard J. Auchus, Sergei G. Tevosian

**Affiliations:** 1 Division of Endocrinology & Metabolism, University of Florida, Gainesville, FL 32610, USA; 2 Malcom Randall VAMC, Gainesville, FL 32608, USA; 3 Department of Physiological Sciences, University of Florida, Gainesville, FL 32603, USA; 4 Department of Genetics, The University of Texas MD Anderson Cancer Center, Houston, TX 77030, USA; 5 Department of Bioinformatics, University of Florida, Gainesville, FL 32610, USA; 6 Charlie Norwood VA Medical Center, Augusta, GA 30904, USA; 7 Department of Physiology, Augusta University, Augusta, GA 30912, USA; 8 Division of Endocrinology & Metabolism, University of Michigan, Ann Arbor, MI 48109-5624, USA; 9 OncoStemyx, Inc., Palo Alto, CA 94401, USA; 10 Department of Surgery, University of Texas Southwestern Medical Center, Dallas, TX 75390, USA; 11 Hamon Center for Therapeutic Oncology, University of Texas Southwestern Medical Center, Dallas, TX 75390-8593, USA; 12 Department of Cell Biology, University of Texas Southwestern Medical Center, Dallas, TX 75390-9039, USA

**Keywords:** adrenocortical carcinoma, histone deacetylase inhibitor, cell differentiation, gene expression

## Abstract

The human adrenal cortex is composed of distinct zones that are the main source of steroid hormone production. The mechanism of adrenocortical cell differentiation into several functionally organized populations with distinctive identities remains poorly understood. Human adrenal disease has been difficult to study, in part due to the absence of cultured cell lines that faithfully represent adrenal cell precursors in the early stages of transformation. Here, Human Adrenocortical Adenoma (HAA1) cell line derived from a patient’s macronodular adrenocortical hyperplasia and was treated with histone deacetylase inhibitors (HDACis) and gene expression was examined. We describe a patient-derived HAA1 cell line derived from the zona reticularis, the innermost zone of the adrenal cortex. The HAA1 cell line is unique in its ability to exit a latent state and respond with steroidogenic gene expression upon treatment with histone deacetylase inhibitors. The gene expression pattern of differentiated HAA1 cells partially recreates the roster of genes in the adrenal layer that they have been derived from. Gene ontology analysis of whole genome RNA-seq corroborated increased expression of steroidogenic genes upon HDAC inhibition. Surprisingly, HDACi treatment induced broad activation of the Tumor Necrosis Factor (TNF) alpha pathway. This novel cell line we developed will hopefully be instrumental in understanding the molecular and biochemical mechanisms controlling adrenocortical differentiation and steroidogenesis.

## 1. Introduction

In mammals, the adrenal cortex is composed of concentric cellular zones that surround an inner medulla (M) and are anatomically and functionally distinct. Three major zones are distinguished in the human adrenal cortex: (a) zona glomerulosa (ZG), (b) zona fasciculata (ZF), and (c) zona reticularis (ZR). Steroidogenic cells present in these three zones synthesize steroid hormones: mineralocorticoids, glucocorticoids, and androgens, respectively. In the murine adrenal cortex, ZG and ZF can be distinguished, but in contrast to humans, the presence of ZR in mice is controversial (e.g., [1,2,3,4]) and no adrenal androgens are produced. Furthermore, rodent adrenals do not express *Cyp17a1* (a gene encoding 17α-hydroxylase/17,20-lyase), and their ZF cells secrete corticosterone, while the main glucocorticoid produced by human adrenals is cortisol [5]. As a result of these notable dissimilarities, mouse models of several human adrenal diseases have been difficult to establish. A thin capsule (C) that surrounds the gland provides the structural support and serves as a source of cortical stem cells [6,7].

Successful production of essential steroidogenic hormones in the adrenal gland relies on the combination of the universal and unique steroidogenic regulatory proteins. Accordingly, master regulators of steroidogenesis confer the common hormone-producing characteristics, while zone-specific enzymes act as refining molecular coordinators for adrenal cell specificity. Steroidogenic factor 1 (SF1/NR5A1) is the transcriptional master regulator of steroidogenic cell identity in several endocrine organs, including the adrenal gland. *Sf1* expression serves as a molecular marker of steroidogenic cell identity [8]. Mice lacking *Sf1* do not develop steroidogenic cells and fail to form gonads or adrenals [9]. In humans, *SF1* mutations cause adrenal failure and a 46,XY-sex reversal [10,11]. As we described previously, GATA4 and GATA6 transcription factors are necessary for the expression of steroidogenic enzymes (e.g., cytochrome P450 family 11 subfamily A (CYP11A1, cholesterol side-chain cleavage enzyme), steroid 3β-hydroxysteroid dehydrogenase/Δ^5/4^-isomerase type 2 (HSD3B2), cytochrome P450 family 11 subfamily B member 1 (CYP11B1, 11β-hydroxylase), cytochrome P450 family 11 subfamily B member 2 (CYP11B2, aldosterone synthase), and cell surface receptors (e.g., melanocortin receptor type 2 (MC2R, the adrenocorticotropin receptor)) [12,13]. Tissue- and zone-specific gene expression patterns serve as unique molecular signatures for human adrenal cell populations. For example, CYP11B2 required for aldosterone synthesis is expressed by ZG cells, while cells residing in the ZF and ZR express CYP11B1 required for the synthesis of glucocorticoids [5].

Immortalized cell lines are critical tools for understanding disease mechanisms. Unlike many other tissues (e.g., muscle, adipose, breast or colon), few or no cultured cell models that faithfully recapitulate adrenal differentiation states currently exist. The armamentarium currently available for understanding human adrenal differentiation in vitro is patently scarce [14,15]. NCI-H295R, the sole human adrenocortical cell line currently in wide use, was derived from a late-stage aggressive carcinoma [16]. This lineage provides a valid and clinically relevant target for drug therapy. However, these cells are not representative of any specific adrenal lineage and, being late-stage cancer, have limitations for studies of adrenal differentiation and neoplasia.

Epigenetic regulation modulates gene expression through modification of nucleosomes (DNA and histones), without altering the DNA nucleotide sequence. Histone acetylation (hyperacetylation) by histone acetyltransferases results in a relatively open chromatin arrangement that is favorable for DNA transcription. In contrast, histone deacetylases (HDACs) catalyze the removal of the acetyl group from the lysine on the target proteins. Their main function is to balance the acetylation level of histones (and other proteins, most notably transcription factors) by opposing the action of histone acetyltransferases. A total of 18 HDAC enzymes that employ zinc- or NAD+-dependent mechanisms to deacetylate acetyl lysine substrates are known in humans. Small molecules that specifically target these epigenetic regulators have been identified. HDACis (Histone deacetylase inhibitors) are natural and synthetically produced compounds that interfere with HDAC function [17]. Here, we describe a non-secretory SF1-positive human adrenocortical adenoma (HAA) derived cell line HAA-1 from a patient with a ZR tumor, which produced dehydroepiandrosterone sulfate (DHEAS). When these cells were placed in culture, they dedifferentiated and no longer produced hormones. We demonstrate here that, upon treatment with HDACis, HAA1 cells undergo steroidogenic differentiation and highly up-regulate the expression of steroidogenic genes and enzymes. We propose that HAA1 represents an early stage in the differentiation of adrenocortical cells and provide a valuable tissue culture model of adrenal differentiation and disease.

## 2. Results

### 2.1. Developing the Novel HAA1 Cell Line from a Benign Neoplasm

**Figure 1:**
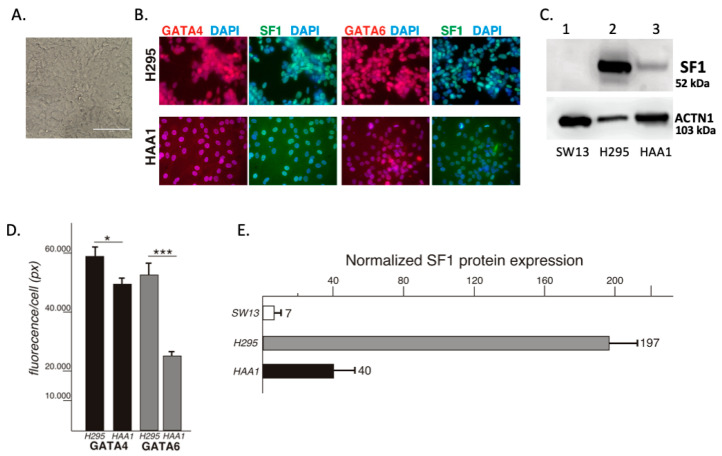
(**A**) HAA1 cells in culture. (**B**) Both master-regulator NR5A1/SF1 (green) and the key adrenal transcription GATA4 and GATA6 proteins (red) are expressed at a lower level in the HAA1 cell line (bottom panels) than in the control NCI-H295R (top panels) human adrenal cortical carcinoma cell line. Scale bar, 50 µ. (**C**) A representative Western blot analysis of protein expression; NR5A1/SF1 (top panel) and ACTIN1 (bottom panel); L1. SW13 adrenal carcinoma cells (these cells produce no steroids and serve as a negative control) L2. NCI-H295.L3. HAA1 (**D**) Quantitative analysis of GATA4 and GATA6 protein expression for the experiment shown in (**B**); *, *p* < 05; ***, *p* < 0.001. (**E**) Quantitative analysis of NR5A1/SF1 expression for the experiment shown in (**C**). All differences are significant, *p* < 0.05.

The HAA1 cell line has been in culture for over eight years and propagated for over 1000 passage doublings. The cells have an epithelial and cuboidal appearance with variably granular cytoplasm (Figure 1A). Upon addition of 10 μM forskolin, the cell line showed evidence of morphologic changes (rounding up, not shown). This cAMP-dependent protein kinase-mediated characteristic has historically been seen with adrenocortical NCI-H295R cells [18].

### 2.2. HAA1 Cells Exhibit Some Characteristics of the Progenitor Cells of the Adrenal

**Figure 2:**
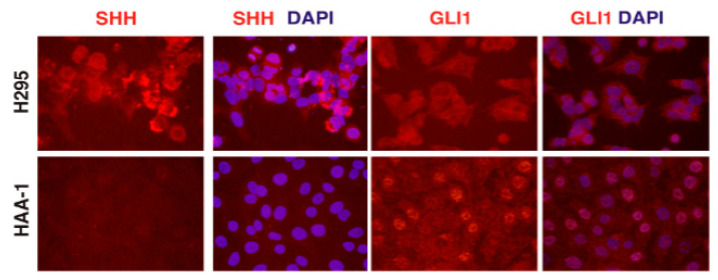
HAA1 cells exhibit some characteristics of the progenitor cells of the adrenal. HAA1 cells (**bottom panels**), but not NCI-H295 cells (**top panels**), express GLI1 protein that is also expressed in the sub-population of the adrenal stem cells, but not SHH. H295 cells express SHH protein. Scale bar, 50 µ.

To characterize the phenotype of the HAA1 cells, we examined the expression of several genes and proteins normally present in adrenocortical cells. Expression of master-regulator SF1 and the key adrenal transcription factors GATA4 and GATA6 in the HAA1 line was present, but lower than that in the control NCI-H295R human adrenal cortical carcinoma cell line (Figure 1B,C). In contrast to the steroidogenic NCI-H295R cells, the HAA1 cell line showed low RNA levels of most steroidogenic enzymes, which were comparable to the non-steroidogenic SW13 cells. However, steroidogenic factor-1 (*SF1, NR5A1*) and *STAR* RNA expression levels in HAA1 cells were intermediate between SW13 and NCI-H295R (Supplementary Figure S1). Since the HAA1 line did not exhibit overt signs of lineage-specific steroidogenic differentiation, we examined the cells for the expression of genes specific to progenitor cells present in the adrenal gland. Sonic Hedgehog (SHH) and GLI1 proteins have been described as markers of the major progenitor cell populations present in the adrenal cortex [7,19,20]. HAA1 cells express nuclear-localized GLI1 protein that is also notably present in the sub-population of the adrenal stem cells [7,20,21,22], but not SHH (Figure 2).

### 2.3. Increased Expression of Master Regulators alone Does Not Induce Re-Differentiation in HAA1 Cells

HAA1 cells retain the expression of master steroidogenic regulators *SF1*, *GATA4* and *GATA6*, but at a lower level than steroidogenically active NCI-H295R cells (Figure 1B,C) and, similar to the progenitor adrenal cells, HAA1 cells express key steroidogenic pathway genes at a low level [23]. One explanation for the absence of steroidogenesis would be de-differentiation and loss of adrenocortical gene expression upon 2D culture conditions over an extended time. Thus, we sought to reestablish steroidogenesis in HAA1 cells. Overexpression of SF1 during embryogenesis is sufficient to cause robust ectopic steroidogenesis in fetal mice [24]. Hence, we tested whether simply boosting the expression of master regulators SF1, GATA4, and GATA6, to the levels of NCI-H295R cells, would be sufficient for re-differentiation. Transient transfection (either separately or combined) of plasmid DNA in which *SF1*, *GATA4* or *GATA6* cDNAs were driven by a strong CMV promoter did not lead to detectable increases in steroidogenic enzyme expression in HAA1 cells (Supplementary Figure S2). Other established approaches for inducing differentiation (e.g., treating cells with the adenylate cyclase activator forskolin or 5-Azacytidine, a DNA methyltransferase inhibitor) were equally unsuccessful.

### 2.4. HAA1 Cells Respond to HDACis by Reprogramming Their Gene Expression

It has been known for a long time that the butyrate ion is a potent inducer of terminal erythroid differentiation in cultured erythroleukemic cells [25]. The mechanistic basis for this phenomenon, based on the ability of butyrate to inhibit histone deacetylation, has been proposed [26]. However, unlike undifferentiated hematopoietic cells that undergo differentiation, cells derived from solid tumors normally respond to HDACis by apoptosis (e.g., [27] and references therein). Nonetheless, we sought to attempt this differentiation-inducing protocol for HAA1 cells. HAA1 cells were treated with various concentrations of sodium butyrate (SoBu), and cells were harvested after 2, 4 or 6 days. RNA was isolated, converted to cDNA, and subjected to qRT-PCR analysis.

**Figure 3:**
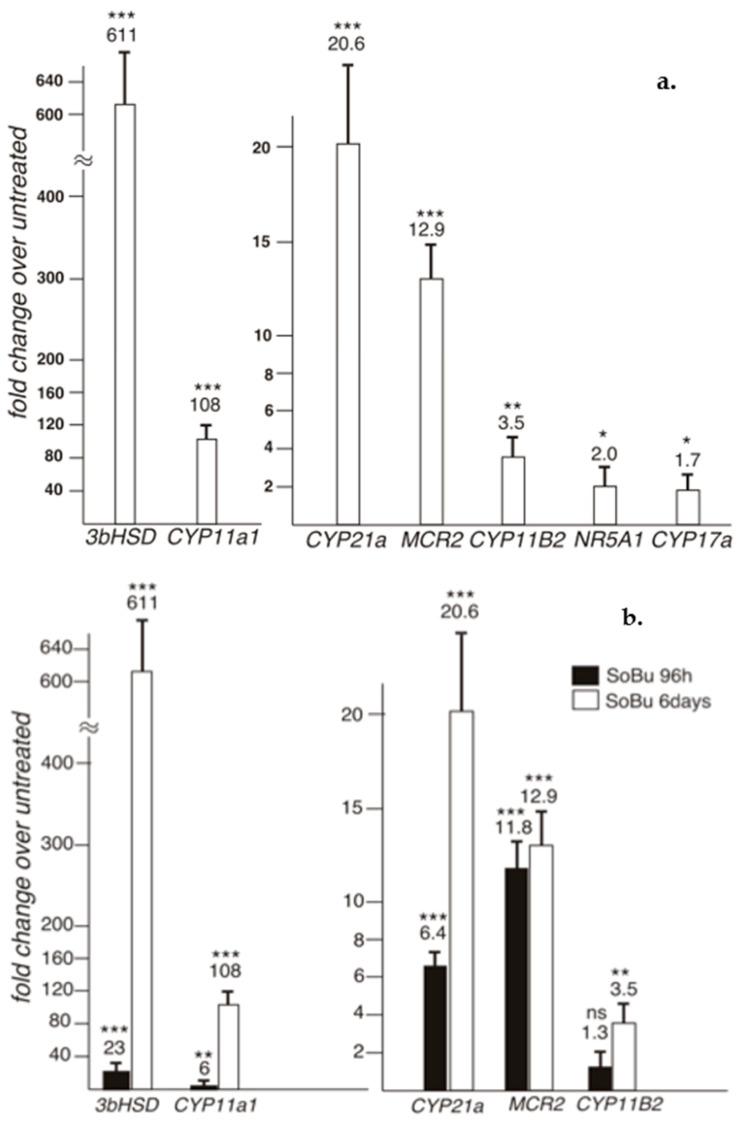

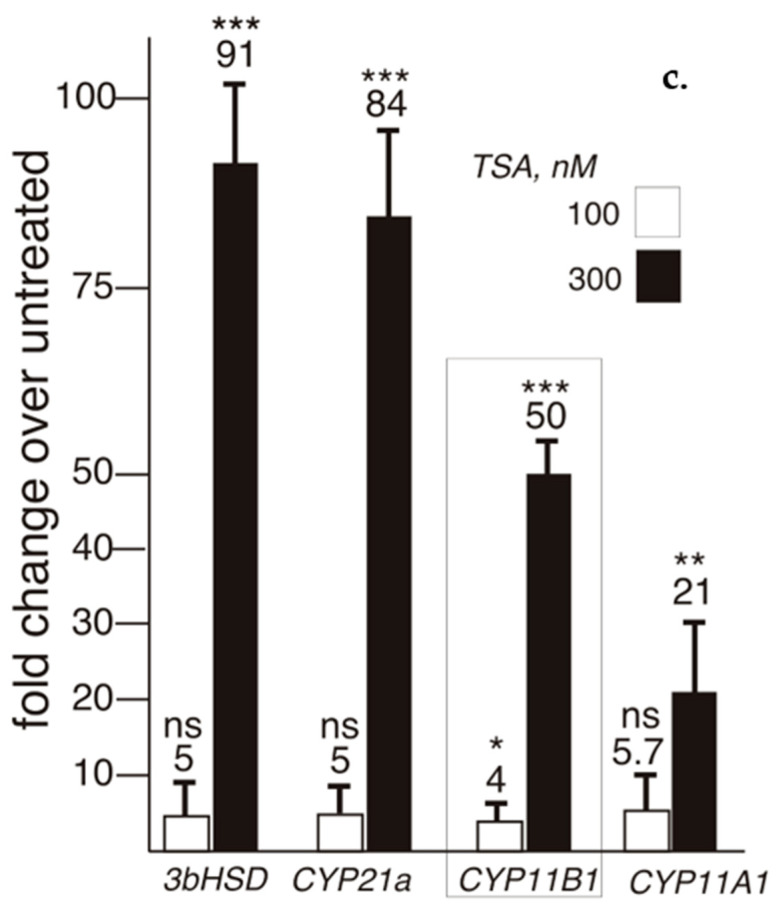
(**a**) qRT-PCR analysis of steroidogenic gene expression in the HAA1 cells. Gene expression in treated cells compared to the untreated is shown. Sodium Butyrate treatment leads to a prominent adrenocortical differentiation in HAA1 cell lines and gene expression for several key enzymes involved in steroidogenic hormone synthesis is highly up-regulated. ***, *p* < 0.001; **, *p* < 0.01; *, *p* < 0.05. (**b**) A comparison of gene expression in HAA1 cells upon 4 (black) or 6 (white) day sodium butyrate treatment. Longer treatment times increase steroidogenic gene expression. ***, *p* < 0.001; **, *p* < 0.01; ns, no significant. The data for 6-day treatment are also shown in 3A. (**c**) qRT-PCR analysis of gene expression in HAA1 cells upon treatment with another HDAC inhibitor, Trichostatin A (TSA). TSA, similarly, is effective in inducing steroidogenic gene expression in the HAA1 cells as sodium butyrate. Notice that CYP11B1 gene expression is efficiently induced in HAA1 cells derived from ZR cells. ***, *p* < 0.001; **, *p* < 0.01; *, *p* < 0.05; ns, no significant.

We determined that SoBu treatment led to a prominent adrenocortical differentiation in HAA1 cells (Figure 3). Gene expression for several key enzymes involved in steroidogenic hormone synthesis was highly upregulated (Figure 3a); longer treatment times resulted in higher gene expression (Figure 3b). Since another HDACi, Trichostatin A, was equally effective in inducing steroidogenic gene expression in the HAA1 cells (Figure 3c), we concluded that it is HDAC inhibition that is capable of promoting steroidogenic re-differentiation in these cells. Interestingly, derived from zona reticularis (ZR), HAA1 cells respond to SoBu by robustly inducing *CYP11B1* mRNA, which encodes the 11β-hydroxylase that catalyzes the final step of cortisol (corticosterone) biosynthesis (Figure 3c) This gene is normally expressed in the ZR and ZF but not the ZG [28]. We also noted that not all zonal-specific steroidogenic gene expression is completely restored in HAA1 cells upon SoBu treatment. For example, CYP17A1 required for the synthesis of adrenal androgens is normally present in human ZR cells. qRT-PCR analysis did not detect a notable increase in *CYP17A1* RNA expression in the cell line upon sodium butyrate or Trichostatin A treatment (Figure 3). In contrast, HDACi treatment of NCI-H295R cells did not result in a robust induction of steroidogenic gene expression (Supplementary Figure S3A).

**Figure 4:**
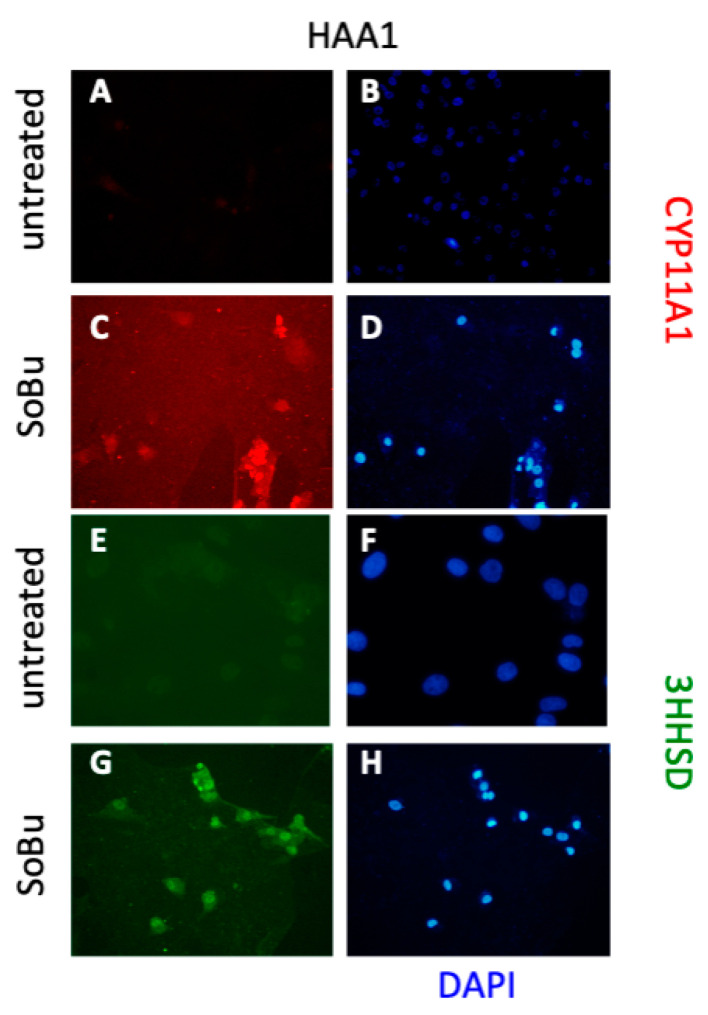
SoBu treatment induces expression of steroidogenic enzymes in HAA1 cells. (**A**–**H**) Untreated (**A**,**B**,**E**,**F**) and SoBu-treated (**C**,**D**,**G**,**H**) HAA1 cells were stained either for CYP11A1 (**A**–**D**) or 3BHSD (**E**–**H**) protein. DAPI staining shows that cells are present in all fields. Scale bars, 100 µ (**A**–**D**,**G**,**H**) and 50 µ (**E**,**F**).

To confirm the RNA expression data at the protein level, we performed immunofluorescence staining analysis using antibodies on CYP11A1 and HSD3B2. CYP11A1 and HSD3B2 immunostaining is prominent only in the HDACi-treated cells (Figure 4). To examine protein expression in HDACi-treated cells in more detail, we performed a Western blot for StAR. The StAR gene encodes for the steroidogenic acute regulatory protein that regulates cholesterol transport within the mitochondria, a rate-limiting step in the production of all steroid hormones. The expression of StAR was elevated upon HDACi treatment in HAA1 cells (Supplementary Figure S4).

### 2.5. Multiple HDACs Contribute to Repression of Differentiation in the HAA1 Cells

**Figure 5:**
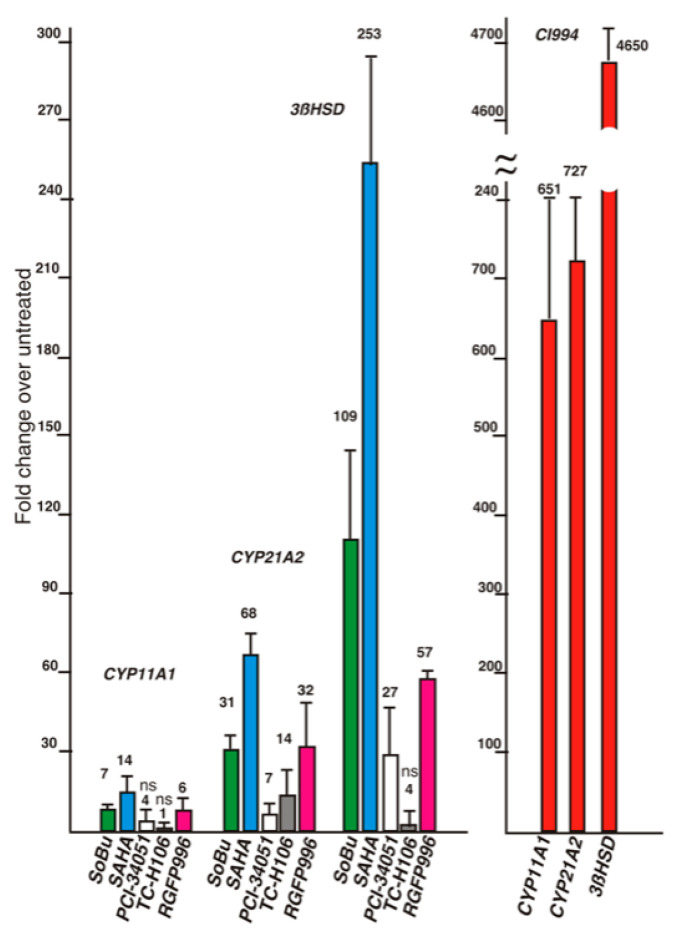
Several HDAC inhibitors were compared for their ability to induce differentiation in the HAA1 cells. CI-994 is the most potent inducer of steroidogenic differentiation in HAA1 cells. All values are significant (*p* < 0.05 or less) except those indicated with “ns”.

Both SoBu and trichostatin A are pan-HDAC inhibitors; therefore, it is not clear which HDAC(s) is/are repressing the differentiation of HAA1 cells. To identify the HDACs involved in this process, we treated HAA1 cells with additional HDAC inhibitors with both broad and selective activity (Figure 5). We noted that, in HAA1 cells, CI994 inhibitor (Selleck Chemicals, Houston, TX, USA), which is reported to inhibit HDACs 1, 3, 6, and 8, is the most effective in inducing steroidogenic differentiation, followed by pan-inhibitors SAHA and SoBu, whereas specific inhibitors PCI-34051 (Selleck Chemicals, Houston, TX, HDAC8-specific) and RGFP966 (Selleck Chemicals, HDAC3-specific) were less potent. Overall, these results suggest that multiple HDACs may contribute to repressing differentiation in the HAA1 cells.

### 2.6. RNA-Seq Analysis of Gene Expression in HAA1 Cells

**Figure 6:**
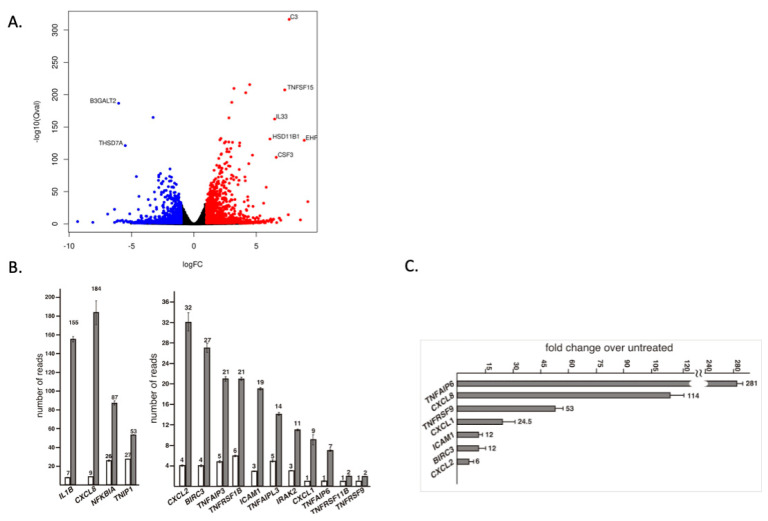
(**A**) A volcano plot of RNAseq analysis of HAA1 cells upon HDACi treatment. The genes with Qval < 4.78 × 10^−95^ and log2 (fold change) > 5 are labeled. (**B**) RNA-seq analysis of gene expression in HAA1 cells after HDACi treatment showing increased expression of a number of TNF-alpha pathway related genes. All values are highly significant, *p* < 0.001. (**C**) qRT-PCR analysis of gene expression in HAA1 cells after HDAC-inhibitor treatment, confirming increased expression of TNF-alpha pathway genes. All values are significant, *p* < 0.05.

To gain a better understanding of the differentiation process in HAA1 cells upon HDACi treatment, we performed an RNA-seq analysis of untreated and SoBu-treated cells (Figure 6A). Ingenuity pathway analysis revealed that the steroidogenic (cholesterol biosynthetic) pathway is the top canonical pathway activated in SoBu-treated HAA1 cells (Table 1).

Additionally, TNF-alpha appears as a top activated regulator (Table 1), with increased expression of numerous inflammation-related genes upon HDACi treatment (Figure 6B). To confirm activation of the TNF-alpha pathway by a different assay, we performed qRT-PCR analysis of several genes associated with this pathway. We demonstrated a profound activation of TNF-alpha pathway genes, confirming the RNA-seq results (Figure 6C). Similarly, the TNF-alpha pathway genes were not induced by the HDACi treatment of NCI-H295 cells (Supplementary Figure S3B).

### 2.7. Chromosomal Characteristics

This is a male-derived cell line with a chromosome number ranging from 45 to 92 and a modal chromosome number of 46. Representative karyotypes of this cell line at passages 25 and 155 are shown in Supplementary Figure S5A,B, respectively. Five consistent clonal markers were present in this cell line. Tentative identification of these markers are M1 = t (8q; ?), M2 = dup (11q), M3 = t (14p; ?), M4 = t (15q; 10q), M5 = t (16q; ?) and M6 = t (17p; 1q). Marker M4 was not present in every metaphase, as shown in Supplementary Figure S5B.

### 2.8. Quantitative Characterization of the Steroidogenic Synthesis in HAA1 Cells upon HDACi Treatment

To examine the steroidogenic potential of the cell line, we measured a set of 23 steroids by LC-MS/MS. We compared the steroid output in media from HAA1 cells under basal conditions and after HDACi treatment using the pan-HDACi SoBu, as well as the optimal HDACi for the cells, CI994. No notable hormone production was observed in untreated or treated cells, despite a dramatic elevation of RNA messages for several key steroidogenic enzymes. To examine the limiting step in steroid hormone biosynthesis in HAA1 cells, we attempted to bypass the first two biosynthetic steps mediated by StAR and CYP11A1. To this end, we supplemented the media with 22(*R*)-hydroxy-cholesterol (22ROH) and pregnenolone. HDACi treatment resulted in the appearance of modest amounts of progesterone in the treated, but not untreated, cells (not shown). We concluded that HDACi-induced differentiation does not induce a complete steroidogenic capacity to a sufficient extent for HAA1 cells to synthesize steroid hormones.

### 2.9. Short Tandem Repeat (STR) Analysis

The HAA1 cells were compared by STR analysis with the H295R (adrenal cortical carcinoma) cell line-ATCC website (https://www.atcc.org/products/all/CRL-2128.aspx#specifications (accessed on 17 October 2019). The HAA1 cells are a unique cell line (Table 2).

## 3. Discussion

In this study, we describe a non-secretory SF1-positive cell line, HAA1, derived from a human DHEAS-producing tumor. We demonstrate that, upon treatment with HDACis, HAA1 cells undergo further steroidogenic differentiation and up-regulate the expression of steroidogenic genes and enzymes. Epigenetic regulation modulates gene expression through the alteration of nucleosomes (by modifying DNA and histones), without changing DNA nucleotide sequence. One of the established regulatory mechanisms is through the control of histone acetylation. The degree of histone acetylation in a cell is mostly determined by opposing activities of two types of enzymes: histone acetyltransferases and HDACs. HDACs’ main function is to balance the acetylation level of histones by opposing the action of histone acetyltransferases. HDACs catalyze the removal of the acetyl group from lysines on target proteins. Intuitively, one may think that HDACs, by promoting chromatin condensation, should be dedicated to gene repression; however, recent evidence points to HDAC function in highly transcribed genes, where they regulate the turnover of acetylated histones and reset chromatin after transcription [29]. On the other hand, histones are not the sole target of HDAC action and hypoacetylation can certainly result in down-regulation of gene expression, and HDAC-dependent down-regulation of key tumor suppressor genes, such as gatekeepers *TP53* and *RB1*, has been reported [30,31]. Information available with respect to HDAC expression and function in adrenocortical cells is very limited. It has been previously reported that HDACis inhibit steroidogenesis through ubiquitination and degradation of steroidogenic factor 1 (SF1, NR5A1) in Y-1 murine cultured cells [32]; however, as the results reported here demonstrate, this observation does not appear to hold true in the human HAA1 cell line. HDAC function in adrenocortical cells in humans remains to be understood.

HDACis include both natural and synthetically produced compounds that interfere with the function of HDACs [17]. While they have important additional targets, the key substrates for these enzymes are the core DNA histones H2A, H2B, H3 and H4 [33]. Histone acetylation (hyperacetylation) by histone acetyltransferases neutralizes the positive charge of the histone tail and destabilizes binding to the negatively charged DNA. This weakened affinity results in a relatively open chromatin arrangement that is favorable for DNA transcription. Acetylation of nucleosomes residing in the vicinity of transcription start sites (TSSs) is thought to promote the binding of chromatin remodeling factors at promoter regions and/or destabilize chromatin structure [34,35], which may lead to decreased nucleosome occupancy immediately upstream of TSSs and facilitate RNA Pol II binding and transcription [36].

Our data show that HDACis induce expression of steroidogenic genes in HAA1 cells. The results also suggest that multiple HDACs may be involved in the suppression of HAA1 steroidogenesis (Figure 5). Furthermore, HDACi treatment of HAA1 cells demonstrates that they preserve some memory of their cell of origin. We found that *CYP11B1* and *MCR2 MC2R* expression is induced in HAA1 cells (Figure 3B,C). We have also determined that untreated HAA1 cells express GLI1 protein that is normally present in the stem cell population in the adrenal cortex (Figure 2). However, a complete pattern of gene expression corresponding to the original adrenal layer was not restored. For example, CYP17A1 was not expressed in HAA1 cells upon HDACi treatment (Figure 3A).

It is not entirely surprising that a complete roster of lineage-specific expression in HAA1 cells is not restored upon HDACi treatment. For example, some of the genes may require a qualitative or quantitative blend of transcription factors that was not recreated as a result of the treatment, or these genes could be controlled through other pathways. It is also possible that these genes were deregulated in the primary tumors that the HAA1 cells were derived from, as adrenal cancer cells are known to exhibit disorganized steroidogenic gene expression [37]. It has been also postulated that early stage, immature steroidogenesis is a characteristic of adrenal tumors. Exploring methylation patterns and chromatin configuration for genes that failed to re-activate in the HAA1 cells upon treatment should be informative and will be the subject of further studies.

Our data further suggest that one of the functions for HDACs could be regulation and suppression of the TNF-alpha and inflammation pathway in adrenocortical cells. Previous research convincingly demonstrated that TNF-alpha is a potent indirect activator of steroid secretion through its ability to stimulate ACTH production [38]. A dedicated role for the intra-adrenal TNF-alpha pathway has also been proposed based on its presence in adrenal cell lines [39,40] and adrenal tumors [39,41]. The addition of TNF-alpha to HAA1 cells did not result in activation of steroidogenic gene expression, so at this point we can only speculate on the role of this pathway in adrenocortical differentiation of HAA1 cells and whether it is a contributing factor or a bystander in their steroidogenic differentiation. In agreement with previous studies, we favor the hypothesis that a chronically active TNF-alpha pathway could be important for activating steroidogenic gene expression in HAA1 cells. In addition, we observed cell death in HDACi-treated cultures of HAA1 cells, consistent with TNF-alpha’s ability to induce apoptosis [42,43]. TNF-alpha can also be a contributing factor to adrenal cancer development and its drug resistance. In conclusion, we have observed that HDAC inhibition partially restores steroidogenic genes in a human cell line derived from an adrenal adenoma.

## 4. Materials and Methods

### 4.1. The HAA1 Cell Line Derivation

Human Adrenal Adenoma Line 1 (HAA1) cell line was derived from a 29-year-old man incidentally found to have bilateral macronodular adrenal hyperplasia. Hormonal work-up revealed elevated DHEA and DHEAS levels, which normalized following bilateral adrenalectomy [23]. Tissue samples were procured under an IRB-approved protocol after obtaining written informed consent. To derive cell line HAA1, tissue from DHEAS-producing macronodular hyperplasia was surgically excised, minced, dispersed with collagenase and DNAse I, and passaged in ACL-4 medium. Primary HAA1 cells were infected with lentiviruses containing human telomerase reverse transcriptase (hTERT) and the human papilloma virus early genes E6/E7, and cells were selected with G418. The resultant line was characterized with phase-contrast microscopy. Later, passage cells were adapted to grow in RPMI 1640 medium supplemented with 10% fetal bovine serum (FBS).

### 4.2. Ectopic Gene Expression in HAA1 Cells

The pCS2_SF1_IRES_EGFP, pCS2_GATA4 and pCS2_GATA6 plasmids were introduced into HAA1 cells by lipofection using Lipofectamine 3000 (Invitrogen, Waltham, MA, USA). For lipofection, 2 µg of plasmid DNA mix was combined with Lipofectamine 3000 reagent as recommended by the manufacturer and added to the mini-chamber slide (Lab-Tek, Grand Rapids, MI, USA). The cells were incubated with the plasmids for 48 h, washed with phosphate-buffered saline (PBS), and fixed with 4% paraformaldehyde.

### 4.3. Immunocytochemistry

Immunofluorescence analysis of HAA1 cells was performed, essentially as previously described [12]. Briefly, cells were grown on chamber slides (Lab-Tek) and used for plasmid DNA transfection, HDACi treatment or as controls. Cells were gently washed with PBS and fixed with 4% paraformaldehyde or cold methanol for 7 min on ice. Fixed cells were washed twice with PBS, and blocked for 30 min in PBS, 5% bovine serum albumin (BSA), and 0.1% Triton X-100. Cells were incubated with primary antibodies diluted in PBS, 1% BSA, and 0.1% Triton X-100. After 1 h of incubation, cells were washed twice with PBS and incubated with Alexa Fluor-conjugated secondary antibodies diluted in PBS/1% BSA/0.1% Triton X-100 for 1 h. Cells were washed and mounted in medium containing 4,6-diamidine-2-phenylidole-dihydrochloride (DAPI, Vector Laboratories, Newark, CA, USA). The following antibody combinations were used: goat anti-GATA4 antibody (R&D Systems, Minneapolis, MN, USA), followed by donkey anti-goat Alexa Fluor 555-conjugated antibodies (Alexa); rabbit anti-GATA6 antibody (Cell Signaling Technology, Danvers, MA, USA) and rabbit anti-GLI1 antibody (Santa Cruz Biotechnology, Dallas, TX, USA) followed by goat anti-rabbit Alexa Fluor 488-conjugated antibodies (Invitrogen); and goat anti-SHH antibody, goat anti-HSD3B2, and goat anti-CYP11A1 (all Santa Cruz Biotechnology) antibodies followed by donkey anti-goat Alexa Fluor 555-conjugated antibodies (Invitrogen). All primary antibodies were diluted 1:300, and all secondary (conjugated) antibodies were diluted 1:500. Images of cells were obtained and photographed using an Olympus BX-51 microscope and an Olympus DP72 digital camera. Images were overlaid in Photoshop and assembled and labeled in Canvas, CorelDraw or Powerpoint. To quantify the GATA4; GATA6 levels, immunofluorescence staining for GATA4 and GATA6 was converted to grayscale and analyzed in a minimum of 20 cells in four sections. The lasso tool was used for nucleus contouring, and the integrated density immunofluorescence for each nucleus was calculated; the background was subtracted from each image. The Mann–Whitney test was performed, and the data were plotted in Excel and presented as corrected total cell fluorescence (CTCF) for nucleus ± SEM.

### 4.4. Western Blot Analysis

Whole cell lysates were prepared using a sodium deoxycholate lysis buffer. Nuclear protein extracts were prepared using a dual buffer method, with the first buffer containing a detergent and the second containing glycerol. Samples were isolated from well washed cell pellets from control or sodium butyrate-treated HAA1 cells and BJ fibroblasts (negative control), which were flash-frozen and kept at −80 °C. The protein in each sample was measured using a NanoDrop Lite spectrophotometer (ThermoFisher Scientific Inc., Waltham, MA, USA). A 100 µL aliquot was separated from the original sample and boiled with 4X LDS sample buffer (Invitrogen) for 5 min. A total of 30 µg of protein for each sample was loaded and resolved on a 12% SDS-PAGE gel along with a BenchMark Protein Ladder (Invitrogen), followed by electroblotting onto PVDF (BioRad, Hercules, CA, USA) membrane. The membranes were incubated with anti-SF1 antibody (Perseus Proteomics, Tokyo, Japan) followed by horseradish peroxidase (HRP)–conjugated anti-mouse secondary (BioRad), and anti-StAR antibody (Santa Cruz Biotechnology) followed by anti-rabbit HRP secondary antibody (BioRad). The HRP signal was developed using Clarify Western ECL substrate (BioRad) and detected using a Li-Cor scanner and Image Studio Digits version 3.1. Sample loading was confirmed through incubation with anti-beta actin antibody (Novus, Centennial, CO, USA), followed by anti-mouse HRP antibody and ECL development and detection. To quantify the protein levels, the staining was converted to grayscale and the images were inverted. The marquee tool was used for band contouring, and the integrated density for each band was calculated; the background was subtracted from each image. The Mann–Whitney test was performed, and the data were plotted in Excel and presented as mean ± SEM.

### 4.5. Chromosome Analysis

HAA-1 cells were cultured in RPMI 1640 medium supplemented with 10% FBS, and chromosome preparations were made at passages 25 and 155 following the standard air-drying technique. Aged slides were G-banded by Trypsin Giemsa technique. G-banded metaphase spreads were photographed using 80i Nikon Microscope and Applied Spectral Imaging (ASI) Karyotyping system. A minimum of ten metaphases were karyotyped.

### 4.6. HDACi Treatment

HAA1 cells were plated onto 60 mm plates at a density of 8.0 × 10^5^ in RPMI 1640 medium supplemented with 10% FBS. Then, 24 h after plating, cells were either left untreated or treated with sodium butyrate (3 mM, Thermo) or trichostatin A (100–300 nM, Sigma-Aldrich, Saint Louis, MO, USA). Cells were maintained in the presence of the HDACi for a defined number of days, at which time the media was removed and cells harvested directly on the plate with TRI^®^ reagent (Sigma-Aldrich, St. Louis, MO, USA). Experiments with HDAC isoform-specific inhibitors and suberoylanilide hydroxamic acid (SAHA) were performed in a similar fashion, except that HAA1 cells were grown in triplicate in 6-well plates (Corning) and either left untreated or treated for 4–6 days with sodium butyrate, Trichostatin A, SAHA (5 µM, #SML0061, Sigma-Aldrich, St. Louis, MO), CI994 (50 µM, #S2818, SelleckChem, Houston, TX, USA), PC-34051 (50 µM, #S2012, SelleckChem) or RGFP996 (50 µM, #S7229, SelleckChem, Houston, TX, USA). RNA from untreated and treated cells was prepared and analyzed as described below.

### 4.7. Total RNA Extraction, First cDNA Synthesis and Quantitative RT-PCR (qPCR)

Total RNA was isolated with the TRI^®^ reagent (Sigma-Aldrich), following the manufacturer’s recommendations, and treated with DNase I (Roche Diagnostics Corporation, Indianapolis, IN, USA), according to the vendor’s instructions. DNase I-treated RNA was purified with Qiagen Mini columns (Qiagen, Germantown, MD, USA), and the quantity and quality of RNA were determined spectrophotometrically with a NanoDrop Lite spectrophotometer. Equal concentrations of total RNA were reverse transcribed using an M-MLV (Moloney Murine Leukemia Virus) Reverse Transcriptase kit (Invitrogen, Thermo), following the manufacturer’s specifications. Quantitative RT-PCR experiments were performed in an ABI 7500 instrument (Applied Biosystems, Foster City, CA, USA) using SYBR Green PCR master mix (Applied Biosystems) under the following conditions: 40 cycles of 95 °C for 15 s and 60 °C for 1 min in a 2-step thermal cycle, preceded by two initial steps: 2 min at 50 °C and 10 min at 95 °C. The primer sequences are shown in Table 3.

For the initial analysis of the HAA1 cells, standardization was performed relative to cyclophilin A (*PPIA*) RNA, and the expression was compared to that in the NCI-H295R and the non-steroidogenic SW13 cell lines. For the analysis of the HDACi-treated cells, standardization of the qPCR data was performed with the endogenous reference *ACTB* (human beta actin) gene RNA. The samples were analyzed in triplicate from at least 3 biological replicates (independent experiments), and the fold change was calculated using the ∆∆Ct method. Statistical analysis (Student’s *t*-test; two-tailed) was performed on the ∆∆Ct values, and the results were considered significant at *p* < 0.05. The results were graphed as fold-change differences relative to wild-type controls using GraphPad Prism^®^, San Diego, CA (6.02 version) software. Fold change equal to 1 represents no change in gene expression.

### 4.8. RNAseq Analysis

HAA1 cells were grown in triplicate and either left untreated or treated with sodium butyrate (3 mM). After six days, cells were harvested and total RNA isolated as described above. For quality control, RNA concentration was determined on a Qubit^®^ 2.0 Fluorometer (ThermoFisher/Invitrogen, Grand Island, NY, USA). RNA quality was assessed using the Agilent 2100 Bioanalyzer (Agilent Technologies, Inc., Santa Clara, CA, USA). Only total RNA with 28S/18S > 1 and RNA integrity number (RIN) ≥ 7 were used for RNA-seq library construction. The RINs of RNA ranged between 7.5 and 9.4.

RNA library construction was performed at the Interdisciplinary Center for Biotechnology Research (ICBR) Gene Expression Core, University of Florida, and sequencing runs were performed in the NextGen core. RNA-seq library preparation was performed with 2 μL of 1:200 diluted RNA spike-in External RNA Controls Consortium (ERCC; 0.5× of the amount suggested in the ERCC user guide: Cat# 4456740) and 1000 ng of total RNA, followed by mRNA isolation using NEBNext Poly(A) mRNA Magnetic Isolation module (New England Biolabs, catalog # E7490 ) and RNA library construction with NEBNext Ultra RNA Library Prep Kit for Illumina (New England Biolabs, catalog # E7530) according to the manufacturer’s instructions. RNA fragmenting time was adjusted according to the RIN of total RNA. Briefly, 1000 ng of total RNA together with 2 μL of 1/200 diluted ERCC were incubated with 15 μL of NEBNext Magnetic Oligo d(T)25 and fragmented in an NEBNext First Strand Synthesis Buffer by heating at 94 °C for the desired time. First strand cDNA synthesis was performed using reverse transcriptase and random primers, and the synthesis of double-stranded DNA was completed using the second-strand master mix provided in the kit. The resulting double-stranded DNA was end-repaired, dA-tailed and ligated with NEBNext adaptors. Finally, the synthesized libraries were enriched by 13 cycles of amplification and purified by Meg-Bind RxnPure Plus beads (Omega Biotek, Norcross, GA, catalog # M1386). For library quality control and pooling, barcoded libraries were sized on the bioanalyzer, quantitated by QUBIT and qPCR (Kapa Biosystems, Wilmington, MA, catalog number: KK4824). A total of 12 individual libraries were pooled at equal molar value of 20 nM, and a total of 2 lanes of HiSeq 000 were run. Differentially expressed genes were plotted as a volcano plot and the genes with Qval < 4.78 × 10^−95^ and log2 (fold change) > 5 were assigned and labeled. Differentially expressed genes were further analyzed using Illumina Pathways analysis and PANTHER. Differential expression of genes belonging to the TNF alpha pathway was analyzed by qRT-PCR as described above.

### 4.9. Liquid Chromatography—Tandem Mass-Spectrometry (LC-MS/MS) Analysis

For the LC-MS/MS experiment, HAA1 cells were grown in 6-well plates in the RPMI media with 10% FBS. Treatments included 15 μM 22*R*-hydroxycholesterol, 15 µM pregnenolone, 10 µM forskolin, without (control) or with HDAC inhibitor, 50 µM of CI-994, to induce steroidogenic differentiation. On day six of the experiment, the media was replaced with the same media as above, except that FBS was omitted. Then, 1 mL aliquots of the media were collected at 0, 4, 8 and 24 h and frozen at −20 °C. Steroid quantitation of 20 3-keto-Δ^4^ (Δ4) and three 3β-hydroxy-Δ^5^ (Δ5) steroids was performed by LC-MS/MS as described previously [53,54].

### 4.10. Short Tandem Repeat (STR) Analysis

Genomic DNA from HAA1 cells was extracted and DNA profiling was performed using the AmpFLSTR Identifier PCR Amplification kit (Thermo Fisher Scientific) and subsequently analyzed on a 3730XL DNA analyzer (Thermo Fisher Scientific). The kit amplified 15 tetranucleotide repeat loci and Amelogenin gender-determining marker. The results were analyzed using GeneMapper v3.7 (Applied Biosystems, Waltham, MA, USA).

## Figures and Tables

**Table 1:** Ingenuity Pathway Analysis of gene expression in HAA1 cells. Summary of Analysis-HAA1 for Ingenuity.

Top Canonical Pathways		
Name	*p*-Value	Overlap
Superpathway of Cholesterol Biosynthesis	6.72 × 10^−15^	53.6% 15/28
LXR/RXR Activation	1.89 × 10^−12^	20.7% 25/121
Hepatic Fibrosis/Hepatic Stellate Cell Activation	2.10 × 10^−12^	16.6% 31/187
Cholesterol Biosynthesis I	7.59 × 10^−11^	69.2% 9/13
Cholesterol Biosynthesis II (via 24,25, dihydrolanosterol)	7.59 × 10^−11^	69.2% 9/13
Top Upstream Regulators	*p* value of overlap	Predicted activation
TNF	1.47 × 10^−55^	Activated
IL1B	9.44 × 10^−35^	Activated
Cg	6.48 × 10^−34^	Activated
TGFB1	1.32 × 10^−32^	Activated
Beta-estradiol	1.73 × 10^−30^	

**Table 2:** Short tandem repeat profile of HAA1.

	Amelogenin	CSF1PO	D13S317	D16S539	D18S51	D19S433	D21S11	D2S1338
HAA1	X, Y	11	12, 13	9, 11	14, 18	14, 15	27, 32.2	24, 25
	**D3S1358**	**D5S818**	**D7S820**	**D8S1179**	FGA *	**TH01**	**TPOX**	**vWA**
HAA1	14, 18	12	8	10, 14	21, 22, 24	7. 9.3	8, 10	16, 18

*, multiple low-level peaks were also observed.

**Table 3:** qRT-PCR primers.

Gene Name	Primers	Reference
1. *CYP11B1*	CYP11B1_For: GGCAGAGGCAGAGATGCTG CYP11B1_REV: TCTTGGGTTAGTGTCTCCACCTG	[44]
2. *CYP11B2*	CYP11B2_FOR: GGCAGAGGCAGAGATGCTG CYP11B2_REV: CTTGAGTTAGTGTCTCCACCAGGA	[44]
3. *CYP17A1*	CYP17A1_For: TGTGGACAAGGGCACAGAAG CYP17A1_Rev: GGATTCAAGAAACGCTCAGGC	[45]
4. *CYP11A1*	CYP11A1_FOR: AGCTAGAGATGACCATCTTCC CYP11A1_REV: GGCATCAGAATGAGGTTGAATG	[45]
5. CYP21A2	Cyp21a2_FOR: ACCTGTCCTTGGGAGACTAC Cyp21a2_REV: TGCGCTCACAGAACTCCTGGGT	[46]
6. HSD3B2	HSD3B2_FOR: AGAAGAGCCTCTGGAAAACACATG HSD3B2_REV: CGCACAAGTGTACAAGGTATCACCA	[47]
7. NR5A1	NR5A1_For: TGGCTACCTCTACCCTGCCTTTCC NR5A1_Rev: GCCTTCTCCTGAGCGTCTTTCACC	[48]
8. StAR	hStAR For: AAGACCAAACTTACGTGGC hStAR Rev: GTGGTTGGCAAAATCCACC	[45]
9. MC2R	MC2R_For: AGCCTGTCTGTGATTGCTG MC2R_Rev: AGATGACCGTAAGCACCACC	[45]
10. SULT2A1	SULT2A1_For: TGATGTCAGACTATAATTGGTTTGAAGGC SULT2A1_Rev: GGTTATGAGTCGTGGTCCTTCCTTATTG	[49]
11. AKR1C3	AKR1C3_For: GAGAAGTAAAGCTTTGGAGGTCACA AKR1C3_Rev: CAACCTGCTCCTCATTATTGTATAAATGA	[50]
12. CYB5A	CYB5A_For: CCAAAGTTAAACAAGCCTCCG CYB5A_Rev: TGTTCAGTCCTCTGCCATG	[51]
13. CYPA	CYPA_For: TATCTGCACTGCCAAGACTGAGTG CYPA_Rev: CTTCTTGCTGGTCTTGCCATTCC	[52]
14. ACTN	hbAct_For: TCACCATTGGCAATGAGCG hbAct_Rev: TGGAGTTGAAGGTAGTTTCGTG	[45]

## Data Availability

All materials described in this publication are available upon request.

## Supplementary Materials

The following supporting information can be downloaded at: https://www.mdpi.com/article/10.3390/ijms24010584/s1.
