# Supplementary material for: Development of Human Adrenocortical Adenoma (HAA1) Cell Line from Zona Reticularis
Source: Int J Mol Sci. 2022 Dec 29;24(1):584. doi: 10.3390/ijms24010584 (PMC9820690; doi:10.3390/ijms24010584)
Supplement: Supplementary file 1 [file ijms-24-00584-s001.zip › ijms-2029547-supplementary.pdf]

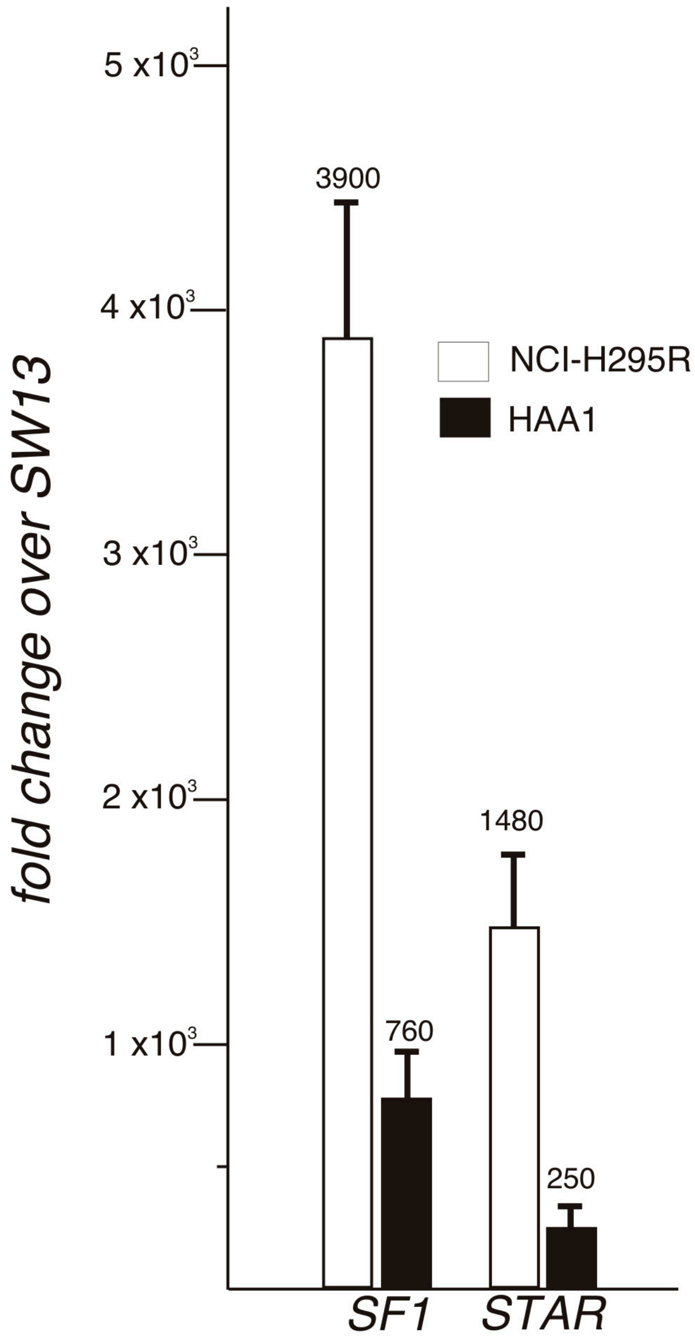

Supp_Figure S1. qRT-PCR analysis of steroidogenic gene expression in the NCI-H295R, SW13 and HAA1 cells. Gene expression in NCI-H295R (white bars) and HAA1 (black bars) compared to SW13 cells is shown. Expression of steroidogenic genes in HAA1 is intermediate between NCI-H295R and SW13 cells. All differences are significant, P<.001.

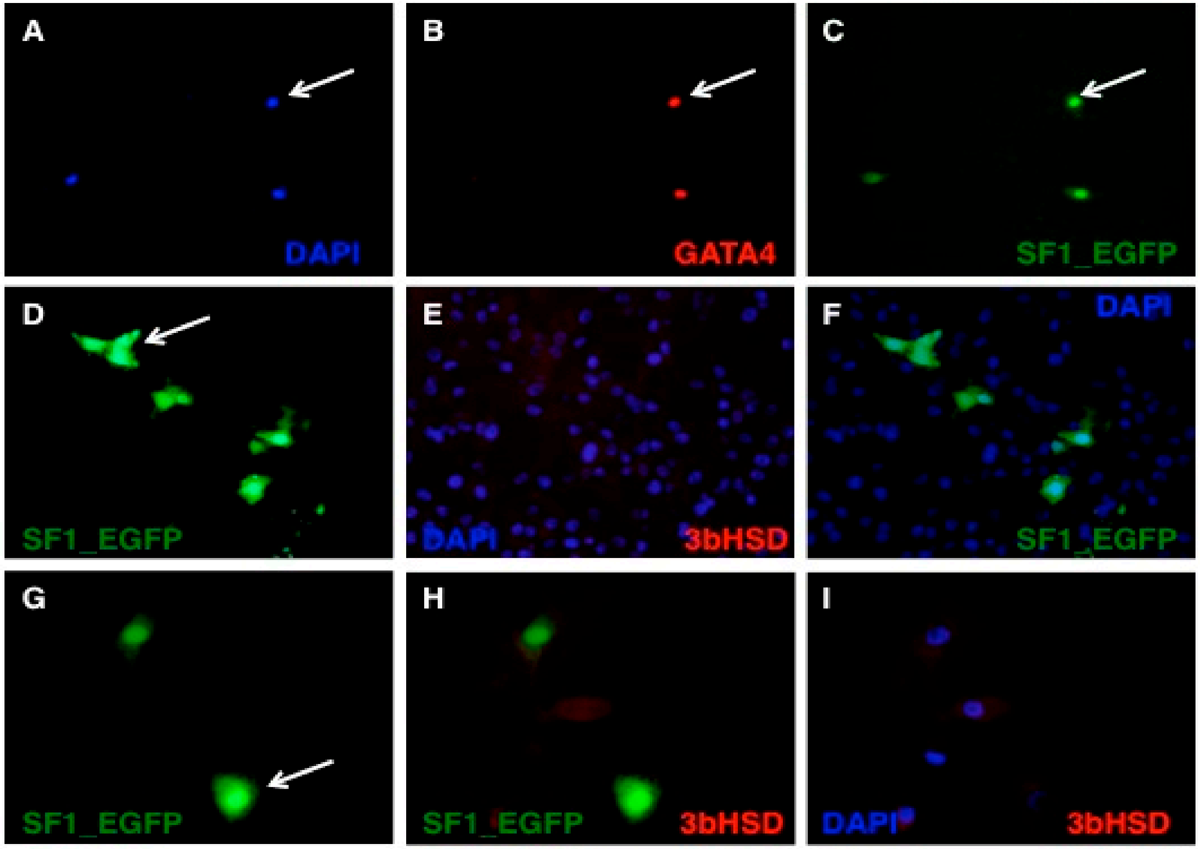

Supp_Figure S2. Transient transfection of master regulators does not induce expression of steroidogenic enzymes. A-C. HAA1 cells co-transfected with SF1_ires_EGFP and GATA4 expressing vectors and stained for DAPI (A) and GATA4 (B). Transfected cells (arrow) express both GATA4 (B) and SF1; EGFP (C) markers. D-I. HAA1 (D-F) cells co-transfected with SF1_ires_EGFP and GATA4 expressing vectors and stained for DAPI (E,F,I) and 3bHSD (E,H,I). Transfected cells (arrow) that should express both SF1 and GATA4 master regulators do not stain positive for the 3bHSD (E,G,H).

A.

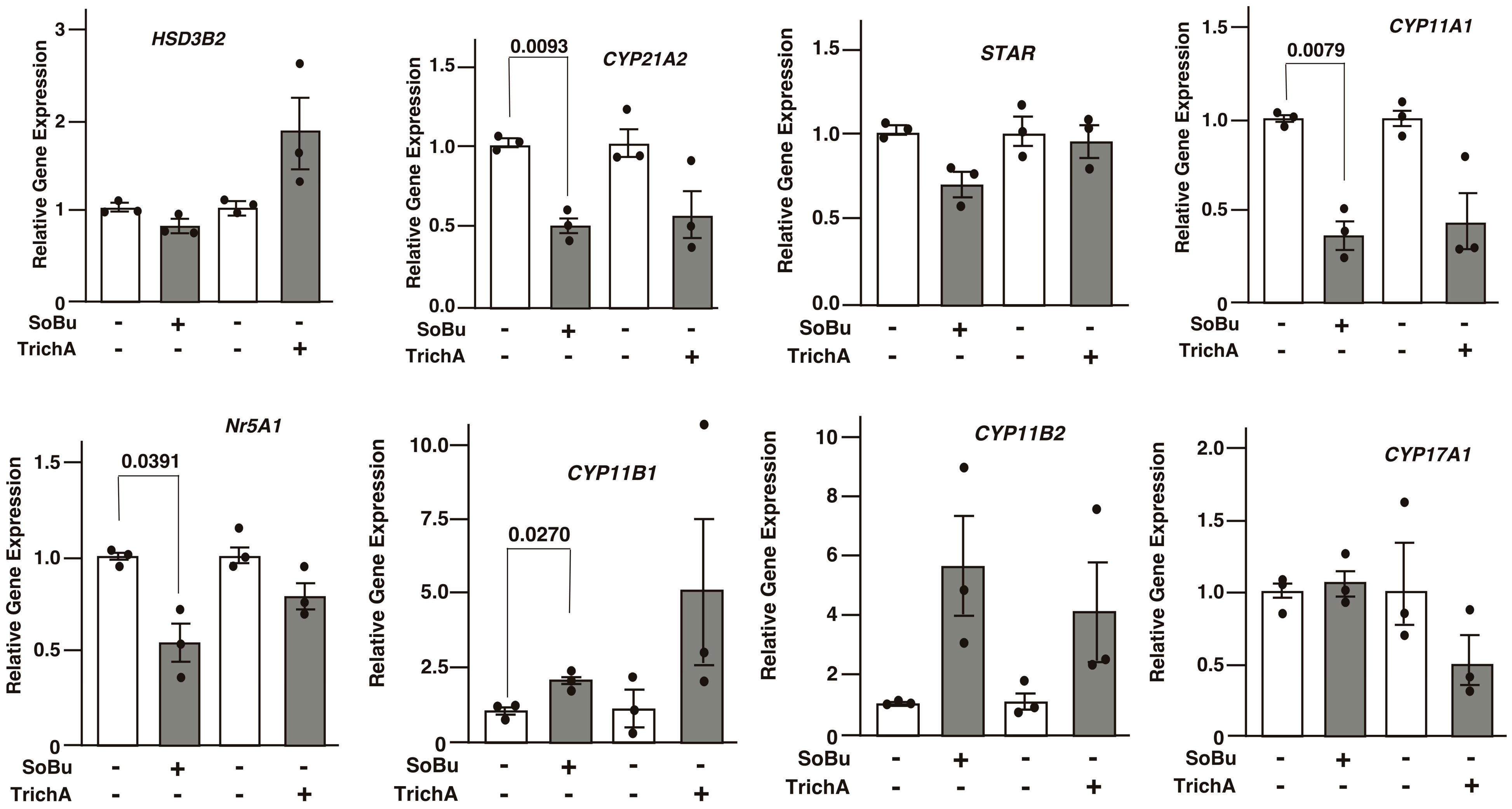

Supp_Figure S3A. qRT-PCR analysis of select steroidogenic (A) or TNF-alpha associated (B) gene expression in NCI-H295 cells upon treatment with HDAC inhibitors, Sodium Butyrate (SoBu) or Trichostain A (TrichA). Relative gene expression in the untreated (*white bars*) compared to HDACi-treated (*black bars*) cells is shown. Differences with P<.05were considered significant as shown.

B.

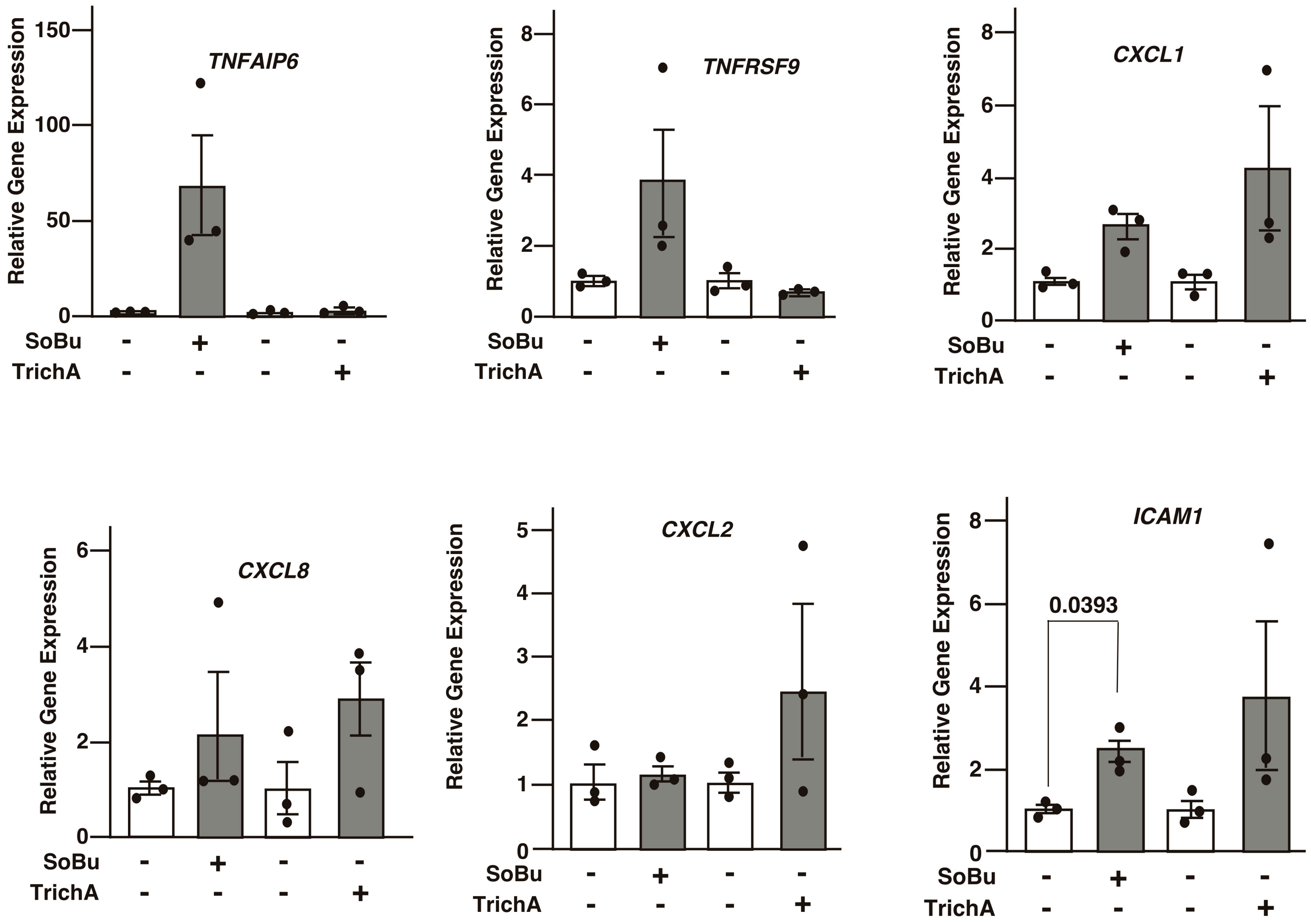

Supp_Figure S3B. qRT-PCR analysis of select steroidogenic (A) or TNF-alpha associated (B) gene expression in NCI-H295 cells upon treatment with HDAC inhibitors, Sodium Butyrate (SoBu) or Trichostain A (TrichA). Relative gene expression in the untreated (white bars) compared to HDACi-treated (black bars) cells is shown.

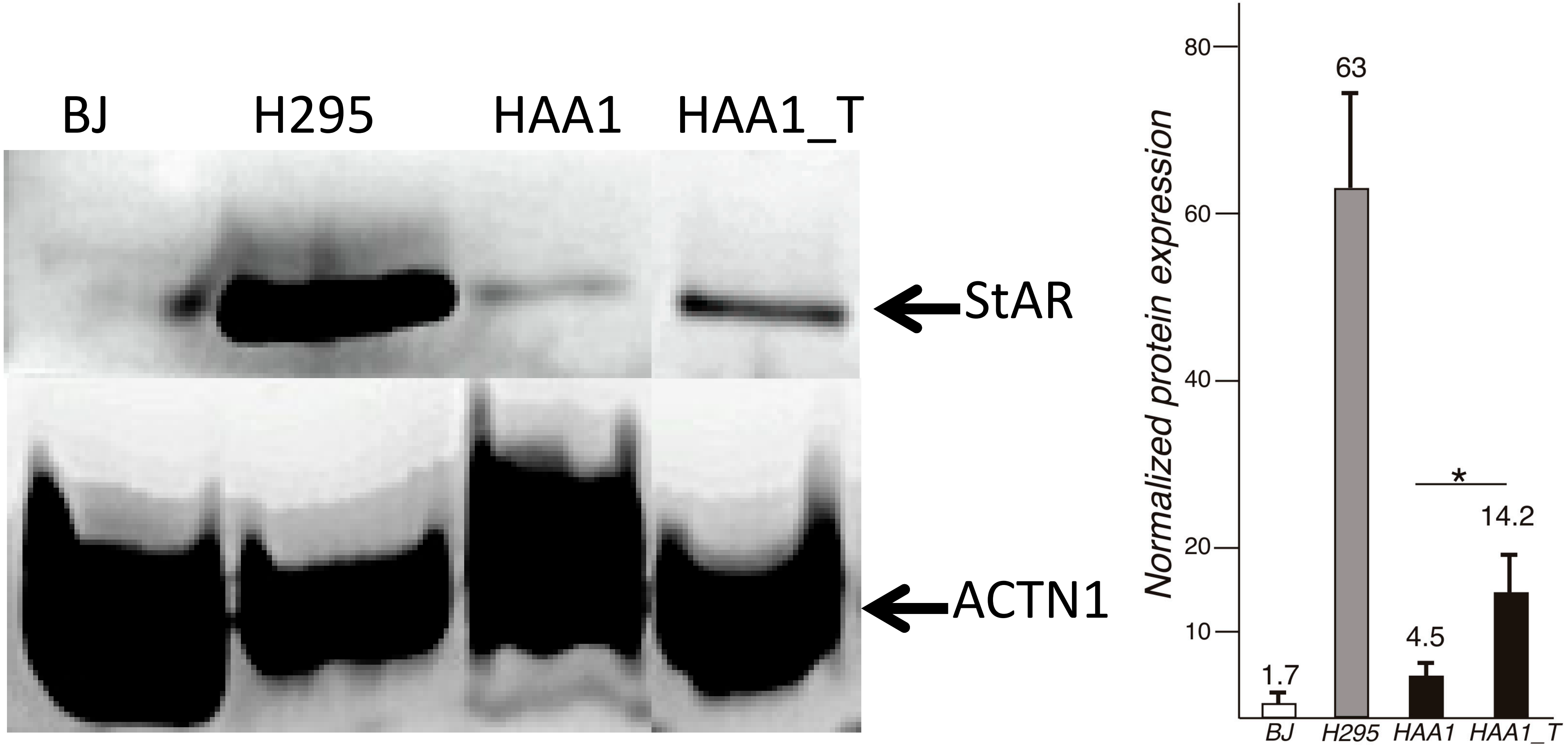

Supp_Figure S4. Western blot analysis of StAR protein expression. StAR levels (top panel) are elevated in treated HAA1 (HAA1_T) cells. StAR expression in positive (NCI-H295) and negative (BJ fibroblasts) control cells is shown. Protein loading in all lanes is confirmed by ACTIN stating (bottom panel).

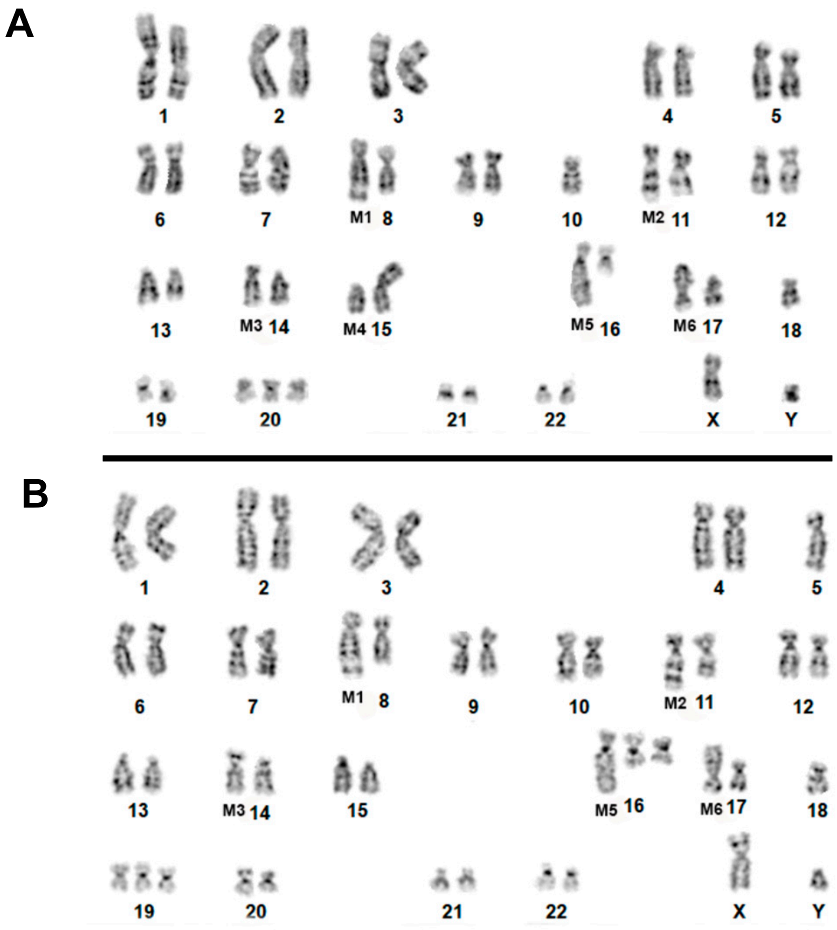

Suppl Figure S5: Representative karyotypes from HAA1 cells at passage 25 (Fig. A) and at passage 155 (Fig. B). For identification of markers, please see the text.
